# The Association between Social Support and Musculoskeletal Health in Community-Dwelling Older Adults: Findings from the Hertfordshire Cohort Study

**DOI:** 10.1007/s00223-024-01307-z

**Published:** 2025-01-03

**Authors:** Gregorio Bevilacqua, Stefania D’Angelo, Faidra Laskou, Elena Zaballa, Nicholas C. Harvey, Elaine M. Dennison

**Affiliations:** 1 MRC Lifecourse Epidemiology Centre, University of Southampton, Southampton General Hospital, Southampton, UK; 2 Victoria University of Wellington, Wellington, New Zealand

**Keywords:** Social support, BMD, Physical capability, Grip strength, Older adults

## Abstract

Previous studies suggest social support is associated with musculoskeletal health in later life. We explored this relationship further in community-dwelling older adults, by considering associations between different aspects of social support and musculoskeletal health in community-dwelling adults. Participants from the Hertfordshire Cohort Study reported level of confiding/emotional, practical, and negative support using the Close Persons Questionnaire. Muscle strength was measured by grip strength dynamometry, and physical capability by timed up-and-go, chair rises, and walking speed tests. Bone mineral density (BMD) was assessed using dual-energy x-ray absorptiometry. Linear regression, adjusted for age, sex, body mass index (BMI), alcohol, smoking, physical activity, social class, and diet, was used for analysis. 1842 men and women (mean age 65.7 years) participated. Low emotional support correlated with weaker grip strength and poorer physical capability tests, although estimates were not robust to adjustment for confounders. Low practical support was linked to shorter timed up-and-go (β − 0.171, 95%CI − 0.319, − 0.024) and walking speed times (β − 0.157, 95%CI − 0.306, − 0.007), following adjustment for confounders. Negative support (i.e. the perceived inadequacy of the support received) was associated with lower grip strength (β − 0.145, 95%CI − 0.223, − 0.067) and slower walking speeds (β 0.159, 95%CI 0.004, 0.314). No social support exposures were associated with BMD. Different types of social support are linked to various measures of musculoskeletal health in older adults. Limited requirement for practical support correlated with better physical capability, while negative support correlated with poorer outcomes. No social support measure was associated with BMD.

**Supplementary Information:**

The online version contains supplementary material available at 10.1007/s00223-024-01307-z.

## Background

The ageing process commonly encompasses a deterioration of the musculoskeletal system typified by the loss of muscle mass (sarcopenia) and bone mass (osteopenia or osteoporosis) [1]. This decline, coupled with reduced physical fitness, can substantially hinder older adults’ ability to perform daily life activities and maintain independence [2]. Reduced physical functioning in later life is common [3], and can have both personal and societal effects, as it leads to increased risk of falls and fractures, as well as inability to self-care [4, 5]. In 2019, around 20% of men and 30% of women in the United Kingdom aged over 65 years required assistance with performing at least one of their daily life activities [6], and it has been estimated that these proportions are likely to increase by approximately a third within the next decade [7]. Poor performance in physical function tests has been previously linked to an increased risk of nursing home and hospital admissions [8]. Maintaining physical function is therefore crucial for older adults and essential from a public health perspective.

Social support describes the physical, financial, and psychological assistance that an individual receives from family members, friends, or the wider community during times of necessity [9]. As such, social support is a complex construct encompassing different types of support, including emotional, informational, practical, or instrumental support [10–12]. Numerous tools have been developed to measure these diverse dimensions of social support [13, 14], each potentially impacting people’s health in different ways. Nonetheless, there is a general consensus that social support can have beneficial effects on various outcomes [15]. Previous studies have suggested that receiving adequate social support may play a protective role for several health outcomes, such as cardiovascular disease [16, 17], hypertension and diabetes [18], depression and anxiety [19], and mortality [20]. In community-dwelling older adults, lack of social support has been linked to decreased quality of life [21].

Previous studies have suggested that overall social support, including emotional support, may be associated with physical capability and health-related behaviours crucial to musculoskeletal health [22, 23], but associations with components of these overall measures are poorly understood. Here, we explored, in a cohort of well-phenotyped community-dwelling older adults in the UK, how distinct dimensions of social support relate to muscle strength, physical capability, and bone mineral density (BMD) to better understand this association.

## Methods

The Hertfordshire Cohort Study (HCS) is a population-based sample of men and women born between 1931 and 1939 in Hertfordshire, UK who still lived there at the time of the study [24]. Participants were initially recruited to investigate the relationship between growth in infancy and risk of adult diseases [24]. A requirement for participation was capacity to provide written consent, and therefore, no individuals with dementia or cognitive impairment were recruited. Between 1998 and 2003, participants completed a nurse-administered questionnaire that collected information on age, marital status, smoking habits, alcohol consumption, physical activity, own current or most recent full-time occupation, and husband’s current or most recent full-time occupation (for ever-married women, used to derive social class). The questionnaire also included a social health section that measured different dimensions of social support. Participants also attended a clinic where their weight, height, handgrip strength, and BMD were measured and where they completed a physical performance assessment.

Clinic visits were conducted country-wide but implemented in phases and organised by geographical area, resulting in some data being collected only for a subset of our sample. For instance, bone scans were collected in East and North Hertfordshire, while chair rise time was measured in North and West Hertfordshire only. This approach has contributed to low completion rates of some measurements in this sample.

### Exposure: Social Support

Social support was assessed using the Close Persons Questionnaire (CPQ), which evaluates the perceived support received from the closest persons over the past 12 months. Three domains were collected: Confiding/emotional support; practical support; and negative support. Confiding/emotional support is a 7-item scale that quantifies the emotional support available from the closest persons. Practical support is a 3-item scale that measures both the need for and receipt of practical help from the closest persons. Negative support is a 4-item scale that captures negative interactions with the closest persons and the perceived inadequacy of the support received. Each item is evaluated on a Likert scale ranging from 1 (“Not at all”) to 4 (“A great deal”). Questions from the CPQ are reported in the Supplementary file (Close Persons Questionnaire).

### Outcomes

During clinic visits, BMD was assessed using a DXA scan of lumbar spine and femoral neck using a Hologic QDR 4500 (Vertec Scientific, Reading, UK). All scans were acquired by a trained technician using standard positioning techniques and in accordance with the manufacturer’s instructions.

Handgrip strength was assessed three times for each hand using a Jamar dynamometer; the maximum measurement was used for analysis [25].

Physical capability was assessed using three Short Physical Performance Battery tests: Walking speed, timed up-and-go, and chair rises [26]. Walking speed was measured using an eight-foot course with no obstructions for an additional foot at either end. Participants walked at their customary pace and the time taken to complete the course was recorded using a stopwatch. Walking speed was determined by dividing the distance traversed by the time between the first and last step.

For the timed up-and-go test, participants had to rise from a chair as quickly as possible, walk three metres at a pace they found comfortable, turn around, walk back to the chair, and sit down again.

For the chair rises test, participants crossed their arms over their chest and stood up from a seated position. Those who could complete this initial task were then asked to stand up and sit down a total of five times. The time taken to complete this was measured with a stopwatch, starting from their initial seated position, and ending when they were standing on the fifth repetition.

### Covariates

Height was measured to the nearest 0.1 cm with a Harpenden pocket stadiometer (Chasmors Ltd, London, UK) and weight to the nearest 0.1 kg on a SECA floor scale (Chasmors Ltd, London, UK). Body mass index (BMI) was calculated as weight divided by squared height (kg/m^2^).

A physical activity time was self-reported (using the Dallosso questionnaire) and calculated as a standardised score ranging from 0 to 100 derived from frequency of gardening, housework, climbing stairs, and carrying loads in a typical week. Higher scores indicated greater levels of activity [27].

Social class was determined using own current or most recent occupation of the participant in men and single women, and of the husband in ever-married women. Occupations were classified as non-manual (classes I-IIINM) or manual (classes (IIIM-V) according to the 1990 OPCS Standard Occupational Classification scheme [28].

The social network score was an indication of the number of people the participant felt close to, while the social activity score was computed with the frequency a participant takes part is a series of social activity ranging from visiting friends, or involvement in clubs and organisations.

Smoking status was self-reported and categorised as never, ex-smoker, or current smoker. Similarly, alcohol consumption was self-reported and measured in units per week, while marital status was also self-reported and analysed as married/cohabiting vs single/divorced/widowed.

## Statistical Methods

Each domain of social support was divided into tertiles [29], with the lowest third of the distribution selected as the reference category in the analyses. The sample for the analysis was restricted to 1,842 participants with all measures of social support. Outcomes were mapped onto a normal distribution (mean of 0 and standard deviation of 1) with Fisher-Yates scores, and therefore, results are expressed as SD change in the outcome for a category change in the exposure. Baseline characteristics of the sample for the overall sample and by tertiles of social support were reported as frequency (%), mean and standard deviation (SD) or median and interquartile range (IQR) as appropriate. Linear regression was used to explore the association between exposure and each outcome. Estimates were first adjusted for age and sex only and then further adjusted for BMI, alcohol consumption, smoking status, physical activity, diet, social class, and marital status. Analyses were performed for men and women combined, as no significant interaction with sex was detected. Our selection of potential confounders was based on pre-existing knowledge of factors that could affect social support and musculoskeletal outcomes without being on their causal pathway. Analyses were conducted with Stata v17.0.

## Results

A total of 1,842 participants provided data on all three domains of social support and at least one outcome, thus were included in the analyses. Specifically, there were 1,610 participants with data available for grip strength, 887 for the 6-m time up-and-go test, 338 for chair rise time, 884 for the 3-m walk time, and 667 for BMD.

Table 1 presents the participants’ characteristics categorised by level of confiding/emotional support. The mean age (SD) of participants was 65.7 (2.8) years. Participants who reported high confiding support were more likely to be male, live with someone, have a better diet, engage in more physical activity, and drink more alcohol (median 7.0, IQR 2.0, 15.9 units per week) compared to those with low confiding support (median 6.0, IQR 1.5, 14.1 units per week). They also reported higher levels of practical support and social network score, while they had lower levels of negative support. The mean grip strength in this sample was 36.5 (11.0) kg, highest among those with high confiding support.

**Table 1:** Baseline characteristics of the sample (*N* = 1842)

		Levels of Confiding/Emotional Support		
	Overall (*n* = 1842)	Low (*n* = 641)	Medium (*n* = 655)	High (*n* = 546)
	Mean (SD), median (lower quartile, upper quartile) or *n*(%)			
Men	1011 (54.9)	333 (52.0)	342 (52.2)	336 (61.5)
Age (years)	65.7 (2.8)	65.7 (2.8)	65.7 (2.7)	65.8 (2.9)
Marital status				
Married/cohabiting	1481 (80.4)	457 (71.3)	547 (83.5)	477 (87.4)
Single/divorced/widowed	361 (19.6)	184 (28.7)	108 (16.5)	69 (12.6)
Prudent diet score	0.06 (1.21)	− 0.04 (1.14)	0.09 (1.24)	0.12 (1.25)
Height, cm	168.4 (9.1)	168.1 (9.2)	168.0 (9.1)	169.1 (8.8)
Weight, kg	77.5 (14.3)	77.3 (14.5)	77.1 (14.3)	78.0 (14.0)
BMI, kg/cm^2^	27.3 (4.3)	27.3 (4.5)	27.3 (4.4)	27.2 (4.0)
Physical activity score (Range: 0–100)	64.3 (50.0, 71.4)	57.1 (50.0,71.4)	57.1 (50.0,71.4)	64.3 (50.0,71.4)
Alcohol consumption, units per week	6.0 (1.5, 14.1)	6.0 (1.5,14.5)	5.0 (1.5,13.1)	7.0 (2.0,15.9)
Smoker status				
Never	847 (46.0)	308 (48.1)	308 (47.1)	231 (42.3)
Ex	781 (42.4)	252 (39.3)	272 (41.6)	257 (47.1)
Current	213 (11.6)	81 (12.6)	74 (11.3)	58 (10.6)
Social class				
I-IIINM	787 (43.5)	289 (45.8)	274 (42.4)	224 (42.1)
IIIM-V	1023 (56.5)	342 (54.2)	373 (57.7)	308 (57.9)
Confiding/emotional support (Range: 0–100)	70.8 (19.4)	–	–	–
Practical support (Range: 0–100)	56.1 (28.1)	39.3 (24.5)	57.5 (24.3)	74.3 (24.4)
Negative support (Range: 0–100)	18.7 (17.1)	21.2 (19.0)	18.1 (15.5)	16.5 (16.3)
Social activity score (Range: 0–100)	43.4 (13.7)	41.8 (13.6)	43.8 (13.7)	44.7 (13.6)
Social network score (Range: 0–100)	60.8 (18.2)	56.9 (18.7)	61.8 (17.5)	64.3 (17.6)
*Outcomes*				
Max grip strength, kg	36.4 (11.0)	35.9 (11.0)	36.0 (10.9)	37.6 (11.0)
6 m time up-and-go (sec)	10.4 (9.3, 11.5)	10.4 (9.5,11.6)	10.2 (9.2,11.5)	10.4 (9.3,11.6)
Chair rise time (sec)	17.2 (14.5, 20.4)	17.4 (14.7,20.9)	17.4 (14.6,20.9)	16.6 (14.1,19.3)
3 m walk (sec)	3.2 (2.9, 3.5)	3.2 (2.9,3.5)	3.2 (2.9,3.5)	3.2 (2.9,3.5)
Baseline Femur BMD (g/cm^2^)	0.803 (0.131)	0.79 (0.13)	0.80 (0.13)	0.82 (0.13)

The sample characteristics stratified by levels of practical support and negative support are detailed in Supplementary Tables 1 and 2, respectively. Participants reporting high levels of practical support shared similar characteristics with participants reporting high confiding support. While participants with high levels of negative support had different profiles and, although more likely to be married, they were more likely to have a poorer diet, lower alcohol intake, and lower social class compared with participants with low levels of negative support. On average they reported lower average confiding score and higher practical support score.

**Fig. 1:**
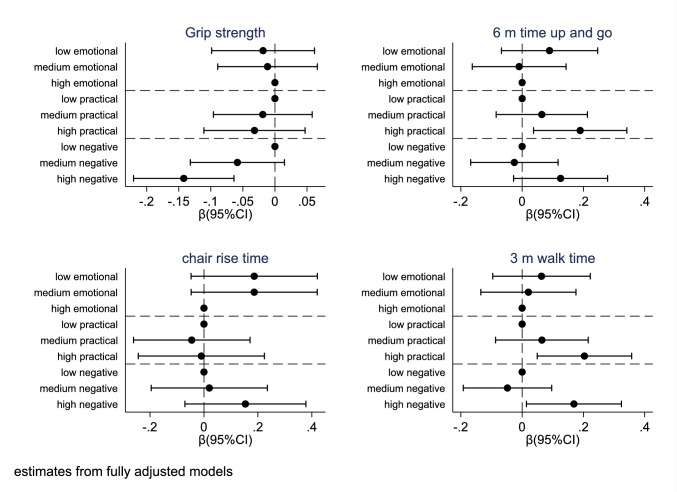
Relationships between different social support dimension and physical functioning measures

**Fig. 2:**
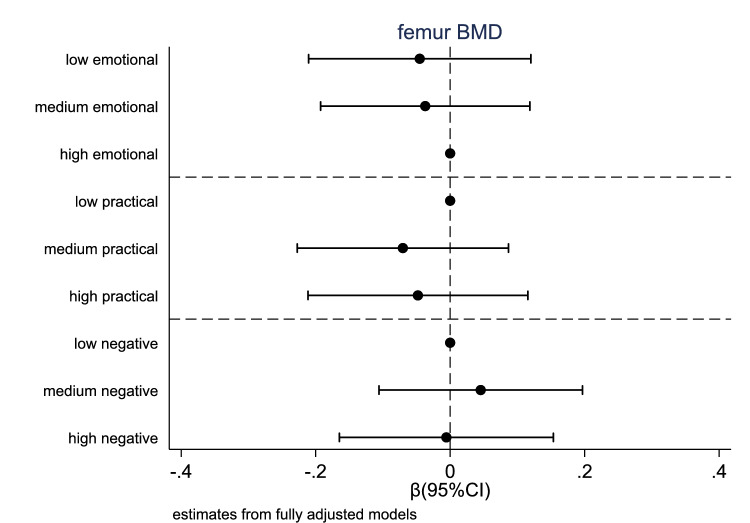
Relationships between different social support dimension and BMD

Figure 1 reports estimates from fully adjusted models. Confiding and practical support were not associated with grip strength once fully adjusted for confounders. However, high negative support (vs low) was associated with lower grip strength (*β* = − 0.145, 95%CI − 0.223, − 0.067). Being in the highest practical support group (vs low) was associated with longer 6-m timed up-and-go (*β* = 0.189, 95%CI 0.037, 0.342) and higher 3-m walk time (*β* = 0.203, 95%CI 0.049, 0.357). Similarly, high negative support (vs low) was associated with higher 3-m walk time (*β* = 0.169, 95%CI 0.014, 0.324). No significant associations were found between any of the domains of social support and chair rise time or BMD (Fig. 2).

## Discussion

In a cohort of UK community-dwelling older adults, we found that different domains of social support were differentially associated with a series of musculoskeletal outcomes. We did not detect significant association between confiding/emotional support and any of the outcomes. However, participants who received higher levels of confiding/emotional support were more likely to be male, have healthier diets, be more physically active, not living alone, and consume more alcohol compared with those receiving low levels of confiding support.

This partly aligns with previous research among Taiwanese older adults, aged approximately 70 years, which found that receiving social support was positively associated with health-promoting behaviours such as physical activity [30]. However, comparison with this study is challenging because social support was assessed using the Health-Promoting Lifestyle Profile Scale [31], which offers a single measure of social support on a four-point Likert scale, rather than distinguishing between specific components of social support. On the other hand, higher alcohol consumption among those receiving greater confiding/emotional support might be attributed to their increased social interactions, as they are more likely to live with others and drink socially, particularly among male participants [32]. This group also reported the highest levels of grip strength in the sample, likely due to their better diets, higher physical activity levels, and predominantly male composition.

We found that receiving more practical support was associated with slower timed up-and-go and walking speed. This appears to be in contradiction with previous studies, which found positive effects of social support on physical functioning [33, 34]. For instance, a longitudinal study including over 800 older adults from the USA, aged 70–79 years, found that participants with higher social support experienced less physical decline over time. Notably, this effect was stronger in male participants [33]. Similarly, a cross-sectional study of 150 community-dwelling Mexican adults aged over 60 years reported a positive relationship between social support and physical functioning [34]. However, comparison with these studies can only be partial, as both studies used tools other than the CPQ to measure social support and employed a physical functioning score derived from multiple tests. In contrast, we focussed on a specific component of the CPQ (i.e. practical support) related to walking speed, a recognized predictive marker of physical functioning among older adults [35, 36], and timed up-and-go. Additionally, the US study had a longitudinal design, whereas ours was cross-sectional. We cannot rule out the possibility of observing a similarly contained decline in our sample if we had longitudinal data. The Mexican study, by contrast, also had a cross-sectional design but was conducted with a small sample of 150 older adults, which may not be representative and limits the generalisability of its findings [34]. These differences may explain the contrasting results of our study. In addition, the association we found between high practical support and poorer physical performance may be a case of reverse causality, as participants with pre-existing poor physical functioning were more likely to receive practical help.

In our population sample, we also found that having negative perceptions of the support received was linked with poor muscle strength and slow walking speed. This was in line with findings reported by Seeman and colleagues in a systematic review looking at health effects of social networks in older adults [37]. Whereas the authors found that social integration has potential health-promoting effects in older adults, they also highlighted how negative social interactions were associated with increased risk of depression and angina [37]. Social support is typically considered as a valuable resource in promoting physical health, but previous research showed that when the support is negatively experienced by those who receive it, this can result in adverse health outcomes [38]. Negative social interactions have been linked to poorer psychological well-being [39], and this can, in turn, lead to reduced motivation to adopt positive health-related behaviours [40], ultimately resulting in reduced physical functioning.

We did not find any association between any of the social support domains and BMD. This contrasts with a study of over 1,800 Korean women aged approximately 70 years, which found that a large social support network was associated with a decreased risk of osteoporosis [41]. While social support is known to be associated with higher engagement in health behaviours which can contribute to BMD maintenance [42], we did not detect an association with BMD in our population sample. This may be due to the cross-sectional design of our study, which prevented us from accounting for the duration of the support received and potential changes in BMD over time. Additionally, our analyses included both men and women, whereas the Korean study focussed exclusively on women, a population with a higher prevalence of osteoporosis [43].

Our study findings must be considered alongside some limitations. Our population sample may not be entirely representative of the wider UK population of the same age, as all participants were born in Hertfordshire, where they were still living in their homes, and were all White. However, it has been previously demonstrated that the HCS is representative of the general population in terms of anthropometric body build and health behaviours (e.g. smoking and alcohol intake) [44]. Although a ‘healthy’ responder bias is evident within the HCS, it has also been shown that this cohort’s characteristics are broadly similar to those in the nationally representative Health Survey for England [24]. In addition, the cross-sectional design of our study may have limited our ability to fully examine possible associations between social support and musculoskeletal health, particularly BMD. Therefore, longitudinal studies are warranted. Moreover, some variables (e.g. smoking status and alcohol consumption) were self-reported in our study and, therefore, recall bias cannot be ruled out. Additionally, for married women, social class was determined at the HCS baseline based on the current or most recent occupation of their husband. This is a crude and outdated assessment that might not accurately reflect the participants’ actual occupation and, therefore, social class, especially among married women. Furthermore, the observational cross-sectional nature of our study means that we cannot make causal influences from the associations, and that although we adjusted comprehensively for possible confounding factors, there is still the possibility of residual confounding and reverse causation. Lastly, the data used in this study were collected over 20 years ago, and our findings may have limited relevance to the present, given the rise digital media and the evolving family dynamics, which now provide alternative and different sources of support.

On the other hand, our study has a number of strengths. We assessed the social support exposure using the CPQ, which has been shown to be a valid and reliable measure of social support [45]. Physical functioning and muscle strength, as well as anthropometric measures, were assessed by a team of trained fieldworkers. These assessments followed relevant procedures and instructions. Lastly, the HCS is a population of community-dwelling older adults that have been extensively phenotyped and well characterised with regard to health-related behaviours and past medical history.

## Conclusions

In a cohort of community-dwelling older adults in the UK, we found that different aspects of social support have distinct associations with musculoskeletal health. We found that lower self-perceived receipt of practical support was associated with better physical capability. Conversely, higher levels of negative support were associated with poorer muscle strength and slower walking speed. However, no significant associations were observed between social support and BMD. These findings highlight the complex interplay between social support and physical health and suggest that the quality of support may be related to physical outcomes more than the mere presence of support. However, the direction of this relationship could not be determined in this cross-sectional study. Further longitudinal studies are needed to better understand these relationships and their long-term implications for musculoskeletal health.

## Supplementary Information

Below is the link to the electronic supplementary material.

Supplementary file1 (DOCX 19 KB)

Supplementary file1 (DOCX 20 KB)

## Data Availability

The datasets used and/or analysed during the current study are available from the corresponding author on reasonable request.
