# Supplementary material for: The Association between Social Support and Musculoskeletal Health in Community-Dwelling Older Adults: Findings from the Hertfordshire Cohort Study
Source: Calcif Tissue Int. 2025 Jan 3;116(1):8. doi: 10.1007/s00223-024-01307-z (PMC11698837; doi:10.1007/s00223-024-01307-z)
Supplement: Supplementary file 1 — Supplementary file1 (DOCX 19 KB) [file 223_2024_1307_MOESM1_ESM.docx]

G. Bevilacqua, S. D’Angelo, F. Laskou, E. Zaballa, N. C. Harvey. E. M. Dennison, *The association between social support and musculoskeletal health in community-dwelling older adults: findings from the Hertfordshire Cohort Study* – Supplementary material: Close Persons Questionnaire

Who have you felt closest to in the last 12 months? Please describe in terms of their relationship to you: (e.g. WIFE, SON, AUNT, BOYFRIEND, MALE FRIEND, FEMALE FRIEND). Remember these are just examples and we would like you to write in whoever you feel closest to.

***Write in the person you are closest to below:-***

Closest

Thinking about the person you are closest to, please tell us how you would rate the practical and emotional support they have provided for you **IN THE LAST 12 MONTHS**

1. How much in the last 12 months did this person give you information, suggestions and guidance that you found helpful?

|  |
| --- |

- 1. Not at all 2. A little 3. Quite a lot 4. A great deal

1. How much in the last 12 months could you rely on this person (was this person there when you needed him/her)?

|  |
| --- |

- 1. Not at all 2. A little 3. Quite a lot 4. A great deal

**c** How much in the last 12 months did this person make you feel good about yourself?

|  |
| --- |

1. Not at all 2. A little 3. Quite a lot 4. A great deal

**d** How much in the last 12 months did you share interests, hobbies and fun with this person?

|  |
| --- |

1. Not at all 2. A little 3. Quite a lot 4. A great deal

**e** How much in the last 12 months did this person give you worries, problems and stress?

|  |
| --- |

1. Not at all 2. A little 3. Quite a lot 4. A great deal

**f** How much in the last 12 months did you want to confide in

(talk frankly, share feelings with) this person?

|  |
| --- |

1. Not at all 2. A little 3. Quite a lot 4. A great deal

|  |
| --- |

**g** How much in the last 12 months **did you confide** in this person? 1. Not at all 2. A little 3. Quite a lot 4. A great deal

G. Bevilacqua, S. D’Angelo, F. Laskou, E. Zaballa, N. C. Harvey. E. M. Dennison, *The association between social support and musculoskeletal health in community-dwelling older adults: findings from the Hertfordshire Cohort Study* – Supplementary material: Close Persons Questionnaire

**h** How much in the last 12 months did you trust this person with your most personal worries and problems?

|  |
| --- |

1. Not at all 2. A little 3. Quite a lot 4. A great deal

**i** How much in the last 12 months would you have **liked to have**   **confided more in this person?**

|  |
| --- |

1. Not at all 2. A little 3. Quite a lot 4. A great deal

**j** How much in the last 12 months did talking to this person make things worse?

|  |
| --- |

1. Not at all 2. A little 3. Quite a lot 4. A great deal

**k** How much in the last 12 months did he/she talk about , his/her personal worries with you?

|  |
| --- |

1. Not at all 2. A little 3. Quite a lot 4. A great deal

**l** How much in the last 12 months did you need practical help from this person with major things (e.g. look after you when ill, help with finances, children)?

|  |
| --- |

1. Not at all 2. A little 3. Quite a lot 4. A great deal

**m** How much in the last 12 months did this person give you practical help with major things?

|  |
| --- |

1. Not at all 2. A little 3. Quite a lot 4. A great deal

**n** How much in the last 12 months would you have liked more practical help with major things from this person?

|  |
| --- |

1. Not at all 2. A little 3. Quite a lot 4. A great deal

**o** How much in the last 12 months did this person give you practical

help with **small** things when you needed it? (e.g. chores, shopping, watering plants etc.)

|  |
| --- |

1. Not at all 2. A little 3. Quite a lot 4. A great deal

Confiding/emotional support = a + c + d + f + g + h + k

Practical support = l + m + o

Negative aspects of support = e + I + j + n
