# Supplementary material for: The Association between Social Support and Musculoskeletal Health in Community-Dwelling Older Adults: Findings from the Hertfordshire Cohort Study
Source: Calcif Tissue Int. 2025 Jan 3;116(1):8. doi: 10.1007/s00223-024-01307-z (PMC11698837; doi:10.1007/s00223-024-01307-z)
Supplement: Supplementary file 2 — Supplementary file1 (DOCX 20 KB) [file 223_2024_1307_MOESM2_ESM.docx]

Supplementary Table 1: Characteristics by levels of practical support

|  | Levels of practical support | | |
| --- | --- | --- | --- |
|  | Low (n=744) | Medium (n=541) | High (n=557) |
|  | Mean (SD), median (lower quartile, upper quartile) or n(%) | | |
| Men | 398 (53.5) | 290 (53.6) | 323 (58.0) |
| Age (years) | 65.7 (2.8) | 65.7 (2.7) | 65.8 (2.8) |
| Marital status |  |  |  |
| Married/cohabiting | 498 (66.9) | 468 (86.5) | 515 (92.5) |
| single/divorced/widowed | 246 (33.1) | 73 (13.5) | 42 (7.5) |
| Prudent diet score | -0.01 (1.2) | 0.14 (1.17) | 0.06 (1.23) |
| Height, cm | 168.2 (9.3) | 168.1 (9.0) | 168.8 (9.0) |
| Weight, kg | 77.4 (14.3) | 76.5 (14.2) | 78.5 (14.2) |
| BMI, kg/cm^2^ | 27.3 (4.3) | 27.0 (4.2) | 27.5 (4.5) |
| Physical activity score (range: 0-100) | 57.1 (50.0,71.4) | 64.3 (50.0,71.4) | 64.3 (50.0,71.4) |
| Alcohol consumption, units per week | 5.8 (1.5,14.0) | 5.5 (1.5,14.0) | 6.4 (1.5,15.5) |
| Smoker status |  |  |  |
| Never | 358 (48.1) | 261 (48.2) | 228 (41.0) |
| Ex | 297 (39.9) | 223 (41.2) | 261 (46.9) |
| Current | 89 (12.0) | 57 (10.5) | 67 (12.1) |
| Social class |  |  |  |
| I-IIINM | 337 (46.0) | 229 (43.0) | 221 (40.6) |
| IIIM-V | 396 (54.0) | 304 (57.0) | 323 (59.4) |
| Confiding/emotional support (range: 0-100) | 59.8 (19.0) | 72.6 (15.6) | 83.7 (13.7) |
| Practical support (range: 0-100) | - | - | - |
| Negative support (range: 0-100) | 17.8 (16.8) | 19.2 (17.3) | 19.4 (17.4) |
| Social activity score (range: 0-100) | 42.9 (14.0) | 43.7 (12.7) | 43.7 (14.2) |
| Social network score (range: 0-100) | 58.7 (18.5) | 61.3 (17.9) | 63.2 (17.8) |
|  |  |  |  |
| *Outcomes* |  |  |  |
| Max grip strength, kg | 36.3 (10.8) | 36.2 (10.8) | 36.7 (11.4) |
| 6 m time up and go (sec) | 10.3 (9.2,11.3) | 10.2 (9.3,11.2) | 10.6 (9.7,12.0) |
| Chair rise time (sec) | 17.3 (14.4,20.5) | 17.4 (14.6,21.0) | 16.6 (14.5,19.7) |
| 3 m walk (sec) | 3.2 (2.9,3.5) | 3.2 (2.9,3.4) | 3.2 (3.0,3.6) |
| Baseline Femur BMD (g/cm^2^) | 0.80 (0.13) | 0.79 (0.12) | 0.82 (0.14) |

Supplementary Table 2: Characteristics by levels of negative support

|  | Levels of negative support | | |
| --- | --- | --- | --- |
|  | Low (n=842) | Medium (n=547) | High (n=453) |
|  | Mean (SD), median (lower quartile, upper quartile) or n(%) | | |
| Men | 469 (55.7) | 286 (52.3) | 256 (56.5) |
| Age (years) | 65.8 (2.8) | 65.6 (2.8) | 65.7 (2.8) |
| Marital status |  |  |  |
| Married/cohabiting | 659 (78.3) | 441 (80.6) | 381 (84.1) |
| single/divorced/widowed | 183 (21.7) | 106 (19.4) | 72 (15.9) |
| Prudent diet score | 0.09 (1.21) | 0.09 (1.22) | -0.04 (1.20) |
| Height, cm | 168.2 (9.3) | 168.6 (9.3) | 168.3 (8.6) |
| Weight, kg | 76.7 (14.3) | 77.9 (13.5) | 78.4 (15.1) |
| BMI, kg/cm^2^ | 27.0 (4.3) | 27.4 (4.1) | 27.6 (4.7) |
| Physical activity score (range: 0-100) | 64.3 (50.0,71.4) | 64.3 (50.0,71.4) | 64.3 (50.0,71.4) |
| Alcohol consumption, units per week | 6.3 (1.6,15.3) | 5.6 (1.5,13.6) | 5.3 (1.5,14.0) |
| Smoker status |  |  |  |
| Never | 394 (46.9) | 245 (44.8) | 208 (45.9) |
| Ex | 348 (41.4) | 244 (44.6) | 189 (41.7) |
| Current | 99 (11.8) | 58 (10.6) | 56 (12.4) |
| Social class |  |  |  |
| I-IIINM | 365 (44.0) | 241 (44.8) | 181 (40.9) |
| IIIM-V | 464 (56.0) | 297 (55.2) | 262 (59.1) |
| Confiding/emotional support (range: 0-100) | 72.2 (20.1) | 71.2 (18.4) | 67.6 (18.8) |
| Practical support (range: 0-100) | 55.2 (29.5) | 55.4 (27.6) | 58.7 (26.1) |
| Negative support (range: 0-100) | - | - | - |
| Social activity score (range: 0-100) | 43.3 (13.6) | 44.3 (14.1) | 42.4 (13.2) |
| Social network score (range: 0-100) | 62.0 (18.2) | 60.6 (17.0) | 58.9 (19.4) |
|  |  |  |  |
| *Outcomes* |  |  |  |
| Max grip strength, kg | 37.0 (11.0) | 35.9 (10.9) | 35.9 (11.1) |
| 6 m time up and go (sec) | 10.3 (9.2,11.3) | 10.3 (9.2,11.5) | 10.4 (9.7,11.9) |
| Chair rise time (sec) | 16.9 (14.2,20.4) | 17.1 (14.6,19.9) | 18.0 (14.8,21.0) |
| 3 m walk (sec) | 3.2 (2.9,3.4) | 3.2 (2.8,3.4) | 3.2 (3.0,3.6) |
| Baseline Femur BMD (g/cm^2^) | 0.79 (0.13) | 0.81 (0.12) | 0.81 (0.14) |
